# Evaluation of Benefits and Health Co-Benefits of GHG Reduction for Taiwan’s Industrial Sector under a Carbon Charge in 2023–2030

**DOI:** 10.3390/ijerph192215385

**Published:** 2022-11-21

**Authors:** Pei-Ing Wu, Je-Liang Liou, Ta-Ken Huang

**Affiliations:** 1 Department of Agricultural Economics, National Taiwan University, Taipei 106319, Taiwan; 2 The Center for Green Economy, Chung-Hua Institution for Economic Research, Taipei 106220, Taiwan; 3 Department of Water Resources and Environmental Engineering, Tamkang University, New Taipei City 251301, Taiwan

**Keywords:** benefit transfer, electricity price, input-output, life cycle, impact pathway, social cost of carbon, value of statistical life

## Abstract

The purpose of this paper is to evaluate the monetary GHG reduction benefits and health co-benefits for the industrial sector under the imposition of a carbon charge in Taiwan. The evaluation proceeds from 2023–2030 for different rates of carbon charge for the GHGs by a model of “Taiwan Economic Input Output Life Cycle Assessment and Environmental Value” constructed in this study. It is innovative in the literature to simulate the benefits of GHG reductions and health co-benefits of air pollutions for the industrial sector under the imposition of a carbon charge comprehensively. The results consistently show benefits whether the charge is imposed on the scope 1 and scope 2 GHG emissions or on the scope 1 emissions only. The health co-benefits are on average about 5 times those of GHG reductions benefits in 2023–2030. The average total benefits with the summation of GHG reduction benefits and health co-benefits are 821.9 million US dollars and 975.1 US million US dollars per year, respectively. However, both the GHG reduction benefits and health co-benefits are consistently increasing at a decreasing rate in 2023–2030. The increased multiple for the rate of the carbon charge is higher than the increased multiple of the total benefits and this result shows that the increase of the carbon charge becomes less effective.

## 1. Introduction

The latest available global direct emissions of greenhouse gases (GHGs) announced by the United Nations Climate Change Secretariat, i.e., scope 1 emissions recorded around the world, show that energy supply accounted for 34%, industrial processes and product use for a further 22%, and all other sectors, including road transportation, agriculture, waste, international transportation, building, and land use, land-use change and forest (LULUCF), for the remaining share of 44% in 2016 [1]. The scope 1 emissions are the amount of direct emissions that the emitting entities own and can fully control [2]. In Taiwan, the corresponding direct emissions for energy supply and industrial production accounted for shares of 68% and 15%, respectively, for the same year. However, if scope 2 emissions, the indirect emissions from the generation of electricity, heating and cooling from energy supply, are accounted for then the energy supply uses up 14% and the industrial sector has a share of 49% [3]. This indicates that most of the energy supplies through the use of electricity are transferred to all other sectors and all manufacturers in the industrial sector. It can be seen that scope 1 emissions from industrial processes and product use and energy supply account for more than 80% of the direct emissions of GHGs.

The most recently available corresponding scope 1 emission records in Taiwan indicate that there were 250 million tonnes in 2020 and, of that, 70% were from the energy supply sector, 13% were from industrial processes and product use, and 17% from all other sectors (as for the global categories indicated above) [3]. This clearly shows that the energy supply and industrial production sectors account for the largest share of scope 1 emissions in Taiwan just as in the rest of the world. If, however, all other sectors include the electricity, heating and cooling from the energy supply sector, i.e., the scope 2 emissions of indirect emissions, the emissions from the energy supply sector account for only 14%. This indicates that the counterpart indirect emissions are transferred to the industrial processes and product use sectors and all other sectors. Among these, the industrial processes and product use sectors account for the largest share of electricity, heating and cooling from energy supply [3]. It is known that the industrial processes and product use sectors have the largest shares of scope 1 and scope 2 emissions. All these emissions have GHG reduction targets set for net zero by the middle of this century in many countries and in Taiwan [4,5].

There are many policy instruments for GHG reduction. Among these, a carbon charge, also referred to as a carbon tax, carbon fee, carbon levy, or even climate contribution is regarded as a mild tool for GHG reductions [6,7,8]. The rationale for this type of policy is that consumers have to pay a higher price for related goods or services and the producers have to bear a relatively heavy financial burden. The implementation of a carbon charge can involve scope 1 emissions and/or scope 2 emissions when a determined GHG target is to be pursued. These reductions will not only bring about the benefits of GHGs but will also result in co-benefits. Past studies have reviewed or summarized various aspects of the co-benefits from GHG reductions.

The term co-benefits was first brought up by the Intergovernmental Panel on Climate Change (IPCC) in the third assessment report in 2001 [9]. Co-benefits are also referred to as ancillary benefits, side benefits, secondary benefits, collateral benefits, and associated benefits. The IPCC further defines co-benefits as “The positive effects that a policy or measure aimed at one objective might have on other objectives, thereby increasing the total benefits for society or the environment…Co-benefits are also referred to as ancillary benefits” [10]. The terms co-benefits and ancillary benefits are now used interchangeably. Co-benefits are mainly classified as involving environmental aspects and social aspects and the specific items include the health improvement of human beings, food security maintenance, sustainable development, the maintenance and preservation of ecosystem services and biological diversity, and the progress of technology. Among the various types of co-benefits, the greatest focus is on human health in accordance with the reductions in air pollutants through the reduction of GHGs because they are easily appreciated by people based on their daily experiences. Without a good command of the monetary health co-benefits from the reduction of air pollutants in GHG reductions, it will be difficult to fully account for the monetary measurement of the social cost of carbon (SCC).

Wu [11] comprehensively reviews past studies on all types of co-benefits and has found that the health co-benefits of human beings are the center of attention among all types of co-benefits. Recent reviews [12,13,14] mainly cover the empirical studies of health co-benefits. The evaluation results of the health co-benefits are commonly presented in terms of the number of persons infected by specific diseases or the morbidity probability of specific diseases. There are only a few studies that evaluate the health co-benefits in monetary terms arising from the reductions in GHGs. Examples of such studies include the cement industry [15], the power or energy sector [16,17], fuel switching households [18], and the transportation and energy sectors [19,20]. Some studies evaluate the health co-benefits for the emission reductions of GHGs for a whole country, sub-national regions, or regions in general, and include South Korea [21], Mexico [22], the north-western region of the US [23], California [24], Sweden [25], China [26], and Europe [27,28]. A study conducted by West et al. [29] has evaluated the global scope of GHG emission reductions.

Moreover, there are some recent studies exploring GHG reduction through carbon neutrality or net-zero greenhouse gas emission for a typical technology, such as carbon capture, solar electricity, circular waste management systems, or thermal heating [30,31,32,33]. These changes will not be implemented, however, until the relevant technology is developed to a mature level. Similar explorations regarding the GHGs reductions are sometimes conducted for a specific city, such as New York City [34] and California [35], or at country level such as for New Zealand [36], or globally [37]. These studies, however, do not integrate both sides of the benefits arising from the GHGs reductions, i.e., GHGs reductions benefits and the accompanying air pollution reduction benefits. The GHGs reductions via all types of technology are basically the main concern of these types of studies.

Not only this, some of the past research even reviews all types of co-benefits from GHGs reductions but without conducting empirical examinations. Some other studies examine the health co-benefits for emission reductions of GHGs for a specific sector or manufacturer. More studies investigate the health co-benefits for specific regions, either for sub-national regions, state, the whole country, or globe. For those with empirical investigations, the evaluation of health co-benefits is given emphasis as these co-benefits are easily ignored in the emissions reductions of GHGs. Among the literature reviewed above, Wang, T. et al. [24] is among the few that has evaluated benefits both for emission reductions of GHGs and health co-benefits. The empirical examination was conducted in California. However, the emission reductions of GHGs are achieved by different assumptions instead from the implementation of policy. To the best of our knowledge, there is no study that simultaneously evaluates the benefits of emission reductions of GHGs and their corresponding health co-benefits from the reduction of air pollution. Without commanding the total benefits from the benefits of emission reductions of GHG and their corresponding health co-benefits, there is no way to judge the appropriateness, either its efficiency or effectiveness, of the GHG emissions mitigation policy, such as introducing a carbon charge.

The results of the health co-benefits evaluation are presented mainly in terms of the reduction of specific types of air pollutant for the purpose of reducing GHGs or CO_2_. The policy tools for the above studies in mitigating GHGs or CO_2_ are different. Some use a clean development mechanism (CDM), joint mechanism (JM), or emissions trading system (ETS) such as [25,28]. All other studies use the most common policy tool, i.e., the levying of a carbon tax on the industry sector, the region, the whole nation, or the whole world. They indicate that the limitation of temperature increases through the imposition of policies on GHGs or CO_2_ emissions is essential and becomes an inevitable mission for each country. The evaluation of benefits from the policies implemented is necessary information to inform policy-makers whether the level of the carbon charge has resulted in the expected level of GHGs or CO_2_ emissions. The evaluation of benefits for specific carbon charges can be used to draw comparisons with the abatement cost to determine the appropriate level of GHGs or CO_2_ emissions.

Taiwan is no exception with regard to the trend of GHG or CO_2_ emission reductions, although it is neither a ratified country in the United Nations Framework Convention on Climate Change (UNFCCC) nor does it have any GHG or CO_2_ emission reduction commitments in the Kyoto Protocol. However, since nationally determined contributions (NDCs) were established in the Paris Agreement in 2016 Taiwan has set specific GHG or CO_2_ emission reduction contributions as part of its national mission. The reduction targets established by the Environmental Protection Administration, R.O.C. Taiwan (the Taiwan EPA hereafter) in 2018 set a goal to reach a level of 2% lower than that for 2005 by 2020, 10% lower than that for 2005 by 2025, and 20% lower than that for 2005 by 2030 [38]. The optimal goal is to achieve a 50% emissions reduction compared to that for 2005 by 2050 [39]. Assurances to achieve a net zero target by the middle of this century were reaffirmed at the Glasgow Climate Pact in COP26 hosted by the UNFCCC in 2021. The net zero in 2050 then also became a new GHG or CO_2_ emission reduction target for Taiwan in the long run.

To fill the gap in past research, the purpose of this paper is to construct a “Taiwan economic input-output life cycle assessment and environmental value” (Taiwan EIO-LCA-EV) model to evaluate the GHGs reduction benefits and health co-benefits for the industrial sector under the imposition of a carbon charge in Taiwan. The evaluation will proceed from 2023–2030 assuming that different rates of the carbon charge are levied on GHG emissions. Two scenarios are designed in this study. The first one is where the carbon charges are imposed on 25 manufacturers in the industrial sector for their scope 1 and scope 2 GHG emissions, and the second scenario consists of those imposed on scope 1 GHG emissions only.

The results of the monetary benefits from GHG reductions and the monetary health co-benefits from air pollution reduction along with the GHG reductions are compared for different manufacturers for different levels of carbon charges for each scenario. The results of each scenario can further be observed for the change in these GHG reduction benefits and health co-benefits in 2023–2030, i.e., the year set for the first NDC goal of moving toward net zero in 2050. Although the literature widely discusses the GHG reductions and health co-benefits theoretically and conceptually, there is no study calculating the GHG reduction’s monetization benefits and the health co-benefit of reducing air pollution along with the reductions of GHG emissions for a specific sector under the imposition of a carbon charge. This makes this study distinct from previous studies achieving a comprehensive evaluation of the monetary benefits of GHG emission reductions for the whole sector and the industrial sector concerned here, together with the corresponding monetary health side effects from the reduction of air pollution. Furthermore, the exploration of the effectiveness for the different levels of carbon charge can guide the related agencies in managing the choice of rate level and its change tempo.

## 2. Methodology

### 2.1. The Need to Account for the Health Co-Benefits of GHG Emission Reductions

**Figure 1:**
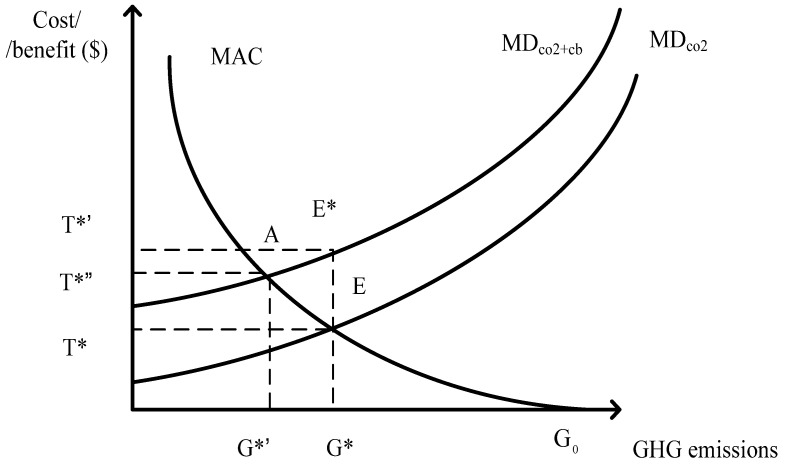
The efficient level of the carbon charge when accounting for health co-benefits.

The marginal damage for GHGs and CO_2_ emissions is MD_co2_. This can be treated as the marginal benefit if GHG and CO_2_ emissions are reduced. For the emission level G*, EG* points to the direct damage and EE* is the indirect damage. The emission level G* generates a total damage of G*E* which is shown as MD_co2+cb_ in Figure 1. If, however, the GHGs emissions G* is eliminated, then EG* is the direct benefit for this emission reduction and EE* refers to the co-benefits of the elimination for the GHGs reductions G*. It is suggested by [40] that if the co-benefit EE* for the emission reductions G* is higher than the marginal abatement cost (MAC) of EG* then it is worth taking action to eliminate emission G* without accounting for the direct benefit EG*. The determination of the carbon charge level when taking into consideration the co-benefits T*’ will be higher than that without T*.

This seems to imply that the imposed target of the carbon charge will not benefit from the existence of GHG co-benefits. It should be explained on the other hand that under a certain level of GHG emissions, the damage not only comes from the emissions of GHGs per se but also from the damage of all other air pollutants that co-exist with the GHG emissions. Thus, in the elimination of a certain level of GHG emissions a higher cost should be borne to reduce the direct damage and indirect damage. In practical terms, as the co-benefits are difficult to measure in monetary terms, the level of the carbon charge T* is determined by MAC and the easy command marginal damage level, i.e., the direct damage EG* only in Figure 1. This carbon charge level is then lower than the actual total damage from the G* level of emissions. Thus, the combination of the monetary measurement of direct benefits with that of indirect co-benefits from certain GHG emission reductions is to ensure that the efficient level carbon charge is achieved.

Once both of the monetary measurement of direct benefits and indirect co-benefits are accounted for, this also has certain implication for the efficient level of carbon charge. The efficient level of carbon charge under such circumstance is T*” and this is higher than that T* when indirect co-benefits are ignored. This will in turn generate more GHGs emissions reductions, G*’, than that when the efficient level of carbon charge without considering the indirect co-benefits shown as Figure 1. It can be concluded that without accounting for the monetary indirect co-benefits the efficient level of GHGs emissions reductions is less than that with co-benefits inclusion. The corresponding efficient level of carbon charge is lower than that with co-benefits containment. Thus, neglecting the indirect co-benefits will provide wrong signal either through the improper level of carbon charge or inappropriate performance of GHGs emissions reductions. The co-benefits include various aspects. The health co-benefit is one of these items. The evaluation of these health co-benefits is surely not complete but this is the first and an important step forward.

### 2.2. Methodology Outline for the Monetary Benefits from GHG Reductions and Monetary Health Co-Benefits from Air Pollution Reduction

In order to achieve the evaluation of monetary benefits from GHG reduction and monetary health co-benefits from air pollution reduction along with the reduction of GHG emissions, a complicated methodology is involved. The related methods in this methodology are outlined here before the detail of each method is presented. The GHG reduction under the imposition of a carbon charge is accomplished by the model of “Economic Input-Output Life Cycle Assessment” (EIO-LCA) suggested by [41,42]. In practice, the EIO-LCA is a combination of the input-output model (IO) and the environmental dimension to reflect the LCA intention. For the case in hand, the environmental aspects include emissions of GHGs and air pollutants, PM_2.5_ and its precursors SO_x_ and NO_x_ [43]. To monetize the benefits both for the GHG reductions and the health co-benefits for air pollution reduction, a model is developed here of “Taiwan Economic Input Output Life Cycle Assessment and Environmental Value” (Taiwan EIO-LCA–EV), an integration of EIO-LCA and the related damage or benefits transferred from the existing studies. All the details of each method mentioned above will be presented in the sequential subsections.

### 2.3. Model Construction for the Emission Reductions of GHGs and Air Pollutants

**Figure 2:**
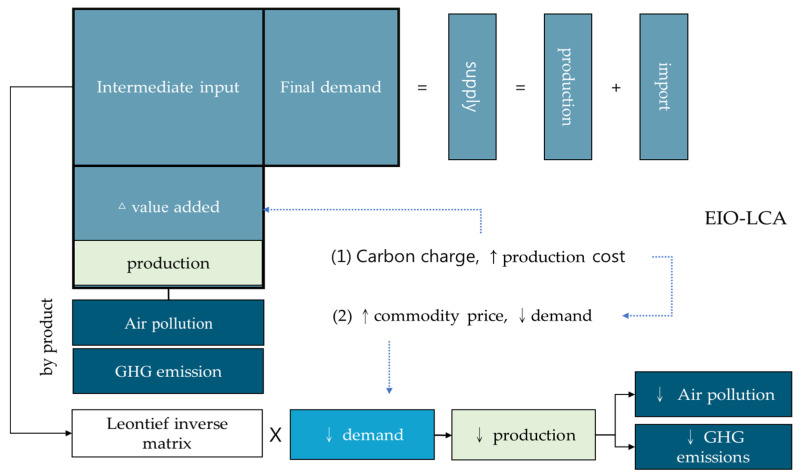
Conceptual framework of the Taiwan EIO-LCA model.

To evaluate the GHG emission reduction for the industrial sector, it is essential to determine the generation of the amount of GHGs in the full life cycle from the production, and utilization to the discarding process for each manufacturer in the sector. The Taiwan EIO-LCA model employed to simulate the emission reductions for GHGs and three air pollutants, i.e., PM_2.5_, SO_x_, and NO_x_, is conceptualized as shown in Figure 2. As the simulation of this study focuses on the aggregate manufacturing level within the industrial level instead of individual products or services, the aggregate manufacturing level for the EIO-LCA is employed to analyze the effects of the carbon charge imposition. The relationship between consumption and production within the EIO-LCA includes the full life cycle of input provision in the upstream industry and the distribution of products in the downstream industry within a specific sector.

In addition, the EIO-LCA also includes the environmental aspects as by products. The analyses of the production side and environmental side are applicable under specific policy implementation. One of the advantages of the EIO-LCA is that there are clear and complete definitions of the evaluation scope for the upstream and downstream data from the input-output table and the environmental statistics from the green gross national product account. This makes the comparison among results meaningful and reliable. As such, the EIO-LCA becomes one of the more popular assessment models at the aggregate sector level [34,35,36,37,38,39,40,41,42,43,44,45,46,47,48,49,50,51,52].

The transaction table in the IO model is composed of the production and the demand (consumption) information dimension. As for the production dimension, the amount of the intermediate input requirements for producing one unit of a specific product and the corresponding payments for each type of input and the amount of indirect tax paid are known. On the demand side, the table indicates the amount used as the production materials for other production units and the final demand for private consumption, investment, government consumption, and net exports, respectively. Based on the demand side, the IO model can be depicted in matrix form as in (1):

(1) $$X={\left(I-A\right)}^{-1}F$$

where I is the identity matrix, A is a matrix formed by the technology coefficients $\alpha_{ij}$, ${\left(I-A\right)}^{-1}$ is the coefficient matrix for direct and indirect requirements, also referred to as the Leontief inverse matrix, X is the production vector for a specific manufacturer in the industrial sector, and F is the sum of the final demand.

One can know from (1) the impact for a one-unit change in the final demand of a specific manufacturer on other manufacturers or sectors through their upstream and downstream production and consumption relationship. The formation of the EIO-LCA involves an integration of environmental by-products with IO shown as (2) [45,46]:

(2) $$E=R\cdot X=R\cdot {\left(I-A\right)}^{-1}F$$

In (2),E is an environment impact matrix generated by the economic production behavior. R is a diagonal matrix and is used to reflect the environmental impact from one unit of product for each manufacturer.

The carbon charge is a type of indirect tax and can be treated as one value-added item. The imposition of a carbon charge will increase the production cost and will consistently increase the product price. The change in the product price can be simulated in an IO price model as shown in (3):

(3) $$\Delta P={\left(I-A^{\prime }\right)}^{-1}\Delta V$$

where ΔP is the change in the product price under the imposition of a carbon charge; ${\left(I-A^{\prime }\right)}^{-1}$ is a non-singular matrix with intermediate inputs and value added in a transaction table and ΔV is the change in value added.

The method suggested by [53,54] is adopted to estimate the change in demand due to the change in the product price resulting from the imposition of a carbon charge as follows:

(4) $$X^{\prime }=PX/P^{\prime }$$

where $P^{\prime }$ is the change in the product price after the imposition of the carbon charge, $X^{\prime }$ is the amount of consumption after the price change, P and X are the product price and consumption amount before the carbon charge. The other item is the final consumption. The measurement of the environmental impact on the change in the consumption of a specific product from a particular manufacturer due to the imposition of a carbon charge is shown as ΔE in (5):

(5) $$\Delta E=R\cdot {\left(I-A\right)}^{-1}\Delta F=R\cdot {\left(I-A\right)}^{-1}\Delta X$$

The environmental impacts of concern here are the change in GHG emissions and various types of accompanying air pollutants under the imposition of a carbon charge. The carbon charge is planned to be imposed on the industrial facilities or processes with at least 25 thousand tonnes of GHG emissions each year in Taiwan. There are 287 facilities or processes that meet this size constraint. Among these, 256 facilities or processes are engaged in manufacturing in the industrial sector and 31 are facilities or processes in the energy sector. The 256 facilities or processes are classified to involve 25 manufacturers in the industrial sector.

Various kinds of data are required for Taiwan’s EIO-LCA. One is the input output table reflecting the latest economic structures in 2016 prepared by the Directorate-General of Budget, Accounting and Statistics, Executive Yuan, R.O.C., Taiwan every 5 years [55]. Another is the *Republic of China National Greenhouse Gas Inventory Report* [56] and the *Green GDP Report* for air pollution [57]. The others are the *Taiwan Emission Data System* [58] and the *Taiwan Energy Statistics Year Book* [59].

### 2.4. Monetary Aspects of Emission Reductions of GHGs and of Air Pollution

**Figure 3:**
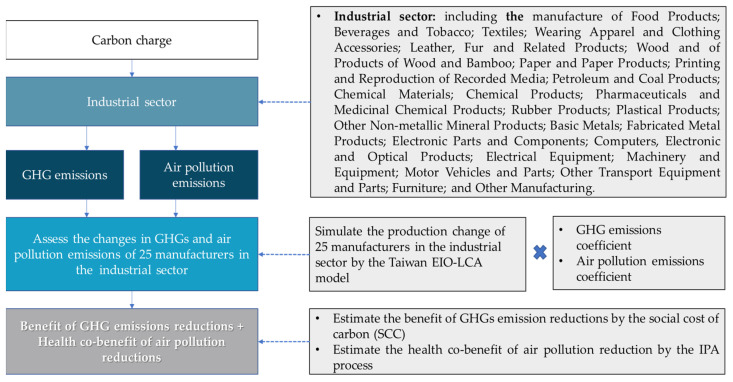
Framework of evaluation of benefits from GHG reduction and health co-benefits of air pollution reduction by means of the Taiwan EIO-LCA-EV model.

The emission reductions of GHGs and the reductions of three air pollutants, PM_2.5_, SO_x_, and NO_x_, are simulated from the Taiwan EIO-LCA model. These reductions can further be measured in monetary terms. Such results are achieved by combining the Taiwan EIO-LCA and the emission reductions for the above environmental components. The framework of the Taiwan EIO-LCA-EV is shown as Figure 3.

The monetization mission is to simulate the benefits of GHG and air pollution reductions for 25 manufacturers in the industrial sector. The benefit evaluations are presented in the following subsections, respectively. The 25 manufacturers include those engaged in food products; beverages and tobacco; textiles; wearing apparel and clothing accessories; leather, fur and related products; wood and products of wood and bamboo; paper and paper products; printing and reproduction of recorded media; petroleum and coal products; chemical materials; chemical products; pharmaceuticals and medicinal chemical products; rubber products; plastic products; other non-metallic mineral products; basic metals; fabricated metal products; electronic parts and components; computers, electronic and optical products; electrical equipment; machinery and equipment; motor vehicles and parts; other transport equipment and parts; furniture; and other manufacturing, respectively [60].

#### 2.4.1. Evaluating the Monetary Benefits of GHG Reduction

One essential piece of information in the evaluation of damage from the emissions of GHGs or benefits from the emission reductions of GHGs shown in Figure 3 by means of the Taiwan EIO-LCA-EV model is the social cost of carbon (SCC). The definition of SCC is the economic cost caused by one ton of GHG emissions or the benefits from one ton of GHGs reductions [61]. The estimation of SCC is an enormous task and involves an extremely high cost. It normally cannot be accomplished by a single or general project. This study is no exception.

The impact of GHG emissions is deemed to be beyond the scope of the country that emits [62,63,64]. Thus, the evaluation results of the SCC obtained by [65,66,67] are suitable for use by all countries. The SCC evaluation by the Working Group involves combining the “Dynamic Integrated Climate-Economy model” (DICE) [68], the “Policy Analysis of the Greenhouse Effect” (PAGE) [69] and the “Framework for Uncertainty, Negotiation and Distribution” (FUND) [70].

This study follows a similar approach to that of many studies in the existing literature by adopting the results accomplished by the Working Group [71,72,73]. The SCC evaluations results delivered by the Working Group under three different discount rates from 2020 to 2050 are presented in Table 1. The selection of the appropriate SCC magnitude for the case at hand requires determination of a discount rate. The discount rate has to take into account the government bond interest rate and the level of the consumer price. An appropriate SCC is chosen based on the above considerations, and the average SCC of under a 2.5% discount rate for 2020 is selected for all the simulations. The monetary benefit from the GHG reductions in the Taiwan EIO-LCA-EV model is then simulated for each manufacturer referred to above.

#### 2.4.2. Evaluation of the Monetary Health Co-Benefits of Air Pollution Reductions

**Figure 4:**
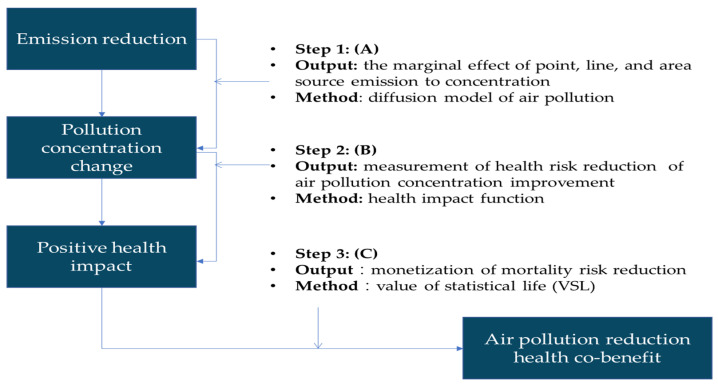
The evaluation of the monetary benefits of air pollution reduction through the IPA.

The monetary evaluation of the health co-benefits from the reductions in GHGs is conducted through impact pathway analysis (IPA) and the framework of the IPA is shown in Figure 4. It is first necessary to know the connection between the amount of air pollution and its corresponding pollution concentration as shown by (A) in Figure 4. We then estimate the reduction in health risk through the change in the morbidity rate and/or mortality rate under each pollution concentration as depicted by (B) in Figure 4. The final step is to transform the morbidity rate and/or mortality rate into monetary terms as shown by (C) in Figure 4. Some of the data used in the IPA to monetize the health co-benefits is from governmental open data, such as the morbidity rate and/or mortality rate for the diseases related to air pollution, and some other data is transferred from the most related studies or parameters for the case in Taiwan as the preferred priority. If no existing studies have been conducted in Taiwan for transfer purposes, cases other than those completed in Taiwan will be transferred as long as an appropriate method of benefit transfer is adopted.

##### The Relationship between Emissions of Air Pollution and Their Concentrations

The relationship between emissions of air pollution involving PM_2.5_, SO_x_, and NO_x_ and their corresponding pollution concentrations is taken from an existing study [74]. The study used Community Multi-scale Air Quality with the Decoupled Direct Method (MAQ-DDM) to simulate the impact of emissions for a specific pollutant and its emission density. The sensitivity regarding the change in emissions can thus be observed. The study reported in [74] provides the effect of one ton of air pollution reductions for each pollutant on the concentration from different sources, i.e., point, line, and area sources as in Table 2. Each pollutant could involve different sources. Thus, the simulation of each pollutant is conducted for each source and the final result is the average of the three sources.

##### Estimations for Contracting Certain Diseases and Emission Concentrations

As with the connection between emission concentration and the contraction of certain diseases, this study uses the most recent version of the environmental benefits mapping and analysis program-community edition (BenMAP-CE) developed by the Environmental Protection Agency of the United States in 2013 and further released to the community in 2015, thereby becoming the BenMAP-community edition (BenMAP-CE). The most recent version of BenMap-CE was published in 2018. The health impact function adopted from the study reported in [75] was used to estimate the change in the health incidence rate as shown in (6) below:

(6) $$\Delta y=\left(1-\exp(-\beta \Delta x)\right)\times y_{0}$$

where $y_{0}$ is the morbidity rate of a particular disease or mortality rate, Δx is the change in pollution concentration, β is the parameter that connects the air pollution and the pollution concentration. All these factors determine the change in the health incidence rate Δy. Among these factors, there is no β parameter available from the studies for Taiwan. Thus, two most commonly used β s from [76], i.e., 0.005827, and from [77], i.e., 0.013103, are adopted in this study. The simulation is performed for each parameter and the final result is the average of the two simulations. The occurrence rate of a specific health incidence is determined by the number of people in the population pop affected by that health incidence and the change in the incidence rate as in (7):

(7) ΔI=Δy×pop

Since the carbon charge imposed on the reduction in GHG emissions is planned for the future, data is then required for the population projection for the years when the carbon charge is implemented. The projected population data is available from [78]. The details for the population each year for 2023–2030 are listed in Table 3.

##### Monetize the Damage from Contracting a Particular Health Incidence

The most essential step is to monetize the damage caused by one ton of air pollution or on the contrary the benefits generated by one ton of air pollution elimination. Thus, in order to estimate the benefit per ton (BPT) for a particular health incidence rate in monetary terms, it is necessary to select the appropriate health incidence to represent the health damage. The health damage can be the medical expenditure for health or the loss of life. The loss of life due to air pollution will be valued at a much higher rate than the medical expenditure. The risk for loss of life is normally used as the cost of air pollution or the benefit from air pollution elimination. Empirically, the value of a statistical life (VSL) is a common method used to estimate the monetary measure for the risk of death. The BPT for the reduction of one ton of air pollution is computed as (8):

(8) $$BPT_{j}=(\Delta I_{j}\times VSL)/P_{j}$$

where $P_{j}$ is air pollutant *j,* and *j* is the PM_2.5_, SO_x_, and NO_x_ of concern here.

The VSL is transferred from other studies. The most recently available VSL conducted in Taiwan is the study reported in [75]. This study uses the value transfer method to adjust the value from [75] to infer the VSL when the carbon charge starts to be implemented in 2023. The VSL value in [75] is the estimated result for the year 2014. The result from [75] is adjusted to 2020 before it is transferred to the forthcoming years after 2020. Assuming the transferred year is *j* years later than 2020, then the transferred VSL for the year 2020 + *j* has to be adjusted as in (9) below. It can be seen that the adjustment is mainly to reflect the wage differences and the wage elasticity.

(9) $$\begin{array}{l} VSL_{2020+j}=VSL_{2020}\times \left\{1+\frac{\left[\in_{w}{}_{{}_{2020}}\times (W_{2020+j}-W_{2020})/W_{2020})\right]}{100}\right\},\;\;\;\;\;\;\;\;\\ \;\;j\;is\;the\;number\;of\;year\;later\;than\;2020 \end{array}$$

The real VSL for the year 2020 + *j* should be adjusted by the consumer price index (CPI) when the carbon charge is implemented as in (10):

(10) $$real\_VSL_{t+j}=VSL_{t+j}\times (CPI_{t+j}/CPI_{t})$$

All parameters are prepared and listed in Table 4 for the VSL transfer.

## 3. Scenario Assumptions and Simulation Results

### 3.1. Principle of Carbon Charge and Emission Allocations among Manufacturers in the Industrial Sector in Taiwan

The carbon charge plan in Taiwan is based on several principles. One is the polluter-pays principle. Thus, the charge target includes direct emissions of scope 1 and indirect emissions of scope 2 to reflect the polluter-pays principle. The principle of imposing a charge on indirect emissions for electricity use in Taiwan is different from that in most of the countries that implement a carbon charge or carbon tax. Facilities and processes with annual emissions of over 25 thousand tons of CO_2_e are the main charge targets. Thus, the building sector and transportation sector are excluded from the imposition of the carbon charge at this stage. According to the inventory data collected by the Taiwan Environmental Protection Administration, there are 287 facilities and processes with scope 1 and scope 2 annual emissions in excess of 25 thousand tons and they are distributed among different types of manufacturing in the industrial sector.

The total direct and indirect emissions for the 256 facilities and processes are about 0.15 billion tons according to the latest inventory data [5] and these amounts account for more than half, i.e., 57.7%, of the total GHG emissions in Taiwan. Among these, the manufacturers with the highest emissions are those involved with chemical materials accounting for 24.2% of emissions, with basic metals accounting for 17.0% in second place, followed by electronic parts and components with a share of 15.1%, non-metallic minerals products with share of 11.8%, and petroleum and coal products with 8.6% of emissions in fifth place. The GHG emissions of these five sets of manufacturers account for 76.7% of total GHG emissions. That is, the top five manufacturers emit about 44% of total GHG emissions in Taiwan. It can thus be observed that the GHG emissions are highly centralized. Moreover, these manufacturers are engaged not only in the traditional energy intensity type but also the electronics type; the manufacturing of optoelectronics and semiconductors is of this type, and has the largest amount of exports, or 172 billion US$ in 2021 [81].

### 3.2. Scenarios Designed for Benefit Simulations of Emission Reductions

The carbon charge is to be implemented at the beginning of 2023 if the legislation is moving smoothly as expected. In accordance with the above principles for the imposition of a carbon charge, the simulated benefits for emission reductions operate under the following assumptions. The beginning of the first period carbon charge is in 2023 and the end is in 2030, when it reaches the first intermediate emission reduction while moving toward net zero in 2050. The charge target in the first stage is for those manufacturers that emit more than 25 thousand tons. The charge rate starts from TWD 100 per ton and consistently increases every other year by TWD 200 per ton in 2023–2030. That is, the rate will be equivalent to US$ 3.3 per ton for 2023–2024, US$ 10 per ton for 2025–2026, US$ 16.7 per ton for 2027–2028, and US$ 23.3 per ton for 2029–2030.

There are two scenarios designed to simulate the benefits of GHG reductions and health co-benefits from air pollution reductions. The first scenario is the carbon charge on the manufacturers with more than 25 thousand tons of emissions annually; these emissions include direct emissions of scope 1 and indirect emissions of scope 2. Under this scenario, the manufacturer is charged based on its direct GHG emissions as well as indirect GHG emissions for the usage of electricity for heating and cooling purposes provided by the energy sector. Although the energy sector does not come within the scope of this study, its emissions through the provision of electricity to the industrial sector are also charged in this scenario as long as the entity’s direct emissions and the emissions from the utilization of electricity exceed 25 thousand tons annually.

The second scenario is that where the carbon fee is charged for direct emissions for those 25 manufacturers in the industrial sector. The manufacturers’ electricity usage in the industrial sector will not be charged the carbon fee under this scenario. The usage of electricity by all manufacturers in the industrial sector is paid through the increase in the cost of the electricity. The energy sector emitted about 0.12 billion tons out of 0.287 billion tons according to the 2019 inventory data (the most recent) for Taiwan [5]. The energy sector is certainly one of the targets of the carbon fee. Among all 0.12 billion tons of emissions from the energy sector, about 93% of emissions involve the provision of electricity to other sectors. Once the energy sector is charged the carbon fee, it is assumed that the rational reaction is to transfer these costs to the electricity price. Under such circumstances, the industrial sector along with all other sectors will pay a higher electricity price.

#### 3.2.1. Results for Scenario 1: The Carbon Charge on Scope 1 and Scope 2 Emissions for 25 Manufacturers in the Industrial Sector

**Figure 5:**
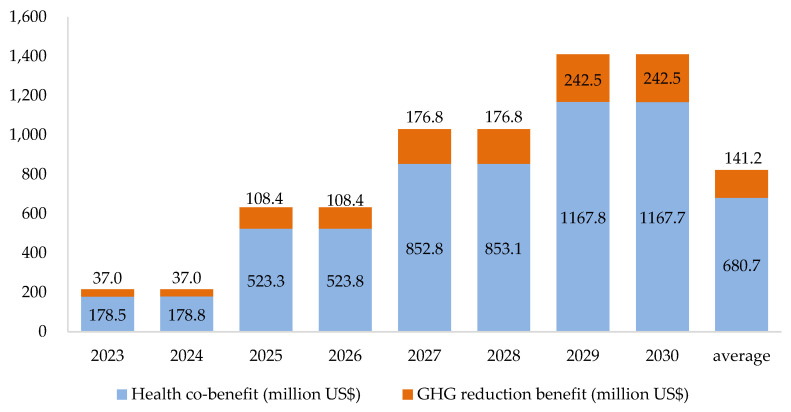
Simulated GHG reduction benefits and health co-benefits for scenario 1 in 2023–2030.

This scenario assumes that the 25 manufacturers in the industrial sector with scope 1 and scope 2 emissions of more than 25 thousand tons per year are charged the carbon fee. These manufactures pay for all the emissions, including the use of fuels directly and the use of electricity indirectly. The simulated results for all the GHG emission reductions for different levels of carbon charge for 2023–2030 are shown in Figure 5. Similarly, the corresponding health co-benefits accompanied by the reductions in air pollution for PM_2.5_, SO_x_, and NO_x_ are also simulated along with the benefits of GHG emission reductions. The results clearly show that the health co-benefits are consistently higher than the benefits from emission reductions for all charge rates. The health co-benefits have slight differences between years with the same charge rate due to the changes in population and wage rates. The benefits from GHG emission reductions, however, are the same as long as the charge rate remains unchanged. On average, the health co-benefits are about 4.82 times those of the GHG emission reductions in 2023–2030.

**Figure 6:**
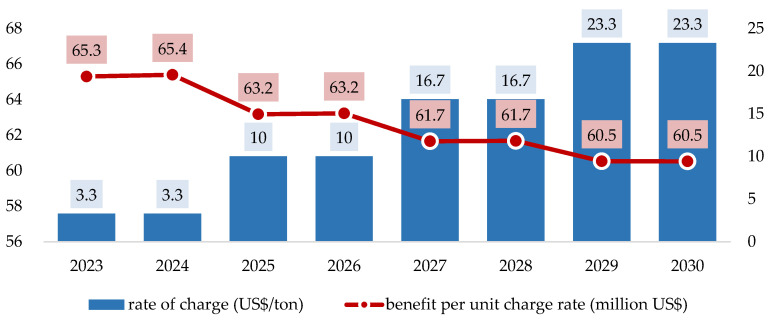
The average benefit for every unit fee rate under scenario 1 in 2023–2030.

Moreover, it is intuitive to see that the higher the charge rate, the higher the benefits from GHG emission reductions. Similarly, the health co-benefits exhibit the same trends. In sum, the total benefits for GHG emission reductions and health co-benefits increase with the rise in the rate of the carbon charge. However, it is observed from the viewpoint of rate efficiency that the increase in the carbon charge rate per US$ will not necessarily generate more benefits. This can be observed by dividing the total benefits at each level of the rate by the rate of the charge per ton. The results are presented in Figure 6. These results indicate that a one US$ increase in the charge rate will generate less total benefits from GHG emission reductions and health co-benefits. This information is essential for GHG control-related agencies. If the effectiveness of the carbon charge policy is not well received by the related agencies, it might create certain obstacles for policy implementation.

Under scenario 1, Table 5 lists the average 2023–2030 total benefits of GHG emission reductions and the corresponding health co-benefits for 25 manufacturers from the highest to the lowest. Among these, the manufacturers with the first five high rankings are the manufacturers of basic metals with total benefits of 40.34% among the 25 manufacturers, manufacturers of other non-metallic mineral products with total benefits of 31.69%, manufacturers of chemical materials with total benefits of 14.34%, manufacturers of petroleum and coal products with total benefits of 3.31%, and manufacturers of paper and paper products with total benefits of 2.36%. These top five manufactures account for 92.04% of the total benefits among the 25 manufacturers. The results indicate that the emissions are highly concentrated among some specific manufacturers. The benefits are certainly highly reliant upon the emission reductions from these manufacturers. The detailed results for the GHG emission reductions and health co-benefits for 25 manufacturers and their share of total benefits are also presented in Table 5.

#### 3.2.2. Results for Scenario 2: The Carbon Charge on Scope 1 Emissions for 25 Manufacturers in the Industrial Sector

**Figure 7:**
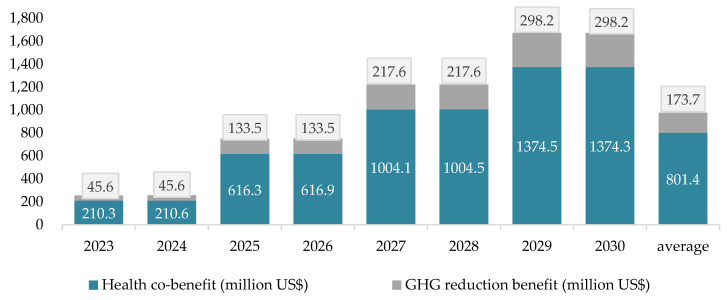
Simulated GHG reduction benefits and health co-benefits for Scenario 2 in 2023–2030.

This scenario is designed to charge a carbon tax on 25 manufacturers. These manufacturers are not charged directly through their use of electricity. However, facilities within the energy sector emitting more than 25 thousand tons of GHG emissions each year are also the target of the carbon charge. Once the GHG emissions from energy facilities and processes are charged a carbon fee it is reasonable for them to transfer these costs to all the end users of electricity. These might include the above 25 manufacturers, all other manufacturers emitting less than 25 thousand tons annually, and all sectors other than the industrial sector. The results of the simulations for scenario 2 are presented in Figure 7.

**Figure 8:**
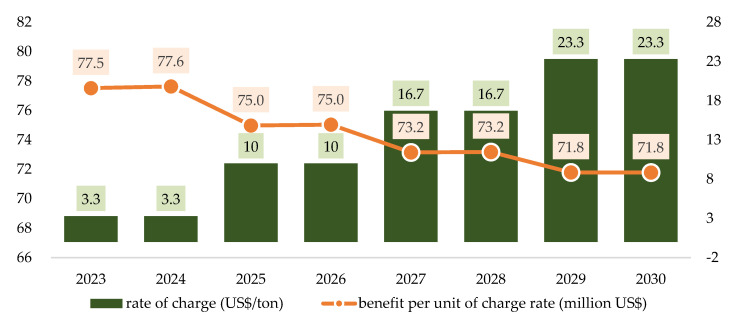
The average benefit for every unit fee rate under scenario 2 in 2023–2030.

Similar to the results for health co-benefits from air pollution reduction, these benefits are higher than their counterpart benefits from GHG emission reductions at each rate of tax charged. The health co-benefits are about 4.62 times the GHG benefits from emission reductions either for individual years or for averages over 8 years. On average, GHG emission reduction benefits and health co-benefits increase as the rate of tax charged increases. This also generates the increasing total benefits along with the increasing rate charged. The benefits from each US$ increase in the charge rate is still decreasing as shown in Figure 8.

It can also be found that GHG emission reduction benefits or health co-benefits or the total benefits from summing up these two are higher than the corresponding benefits simulated in scenario 1 for each charge rate. These results indicate that for the 25 manufacturers of most concern in this study it is less effective in terms of GHG emission reductions if the carbon charge proposed by the government involves charging scope 1 and scope 2 GHG emissions. In other words, the burden for these 25 manufacturers is higher if they are only charged for scope 1 GHG emissions. Their production cost is significantly higher than the amount they pay for being charged based on the scope 2 GHG emissions in scenario 1.

The benefits for the top five manufacturers among these 25 manufacturers are the same as those for scenario 1. The ranking of the total benefits is also the same but the share of benefits of each manufacturer is different from that for scenario 1. The total benefits from manufacturers of basic metals are ranked first and account for 37.91% total benefits for all 25 manufacturers, with manufacturers of other non-metallic mineral products with 29.69% of total benefits in second place, manufacturers of chemical materials with 17.20% of total benefits third, manufacturers of petroleum and coal products with 4.06% of total benefits fourth, and manufacturers of paper and paper products with 2.54% of total benefits fifth. These five manufacturers contributed 91.40% of total benefits from their reductions of GHG emissions. This amount is slightly lower than that in scenario 1.

Although the total benefits for each rate of tax charged in this scenario are higher than those for scenario 1, the top five manufacturers do not concurrently provide higher total benefits from GHG emission reductions. This demonstrates that all the other 20 manufacturers have relatively high GHG emission reductions due to the increase in input prices, i.e., the electricity price. The simulated results for the health co-benefits and GHG reduction benefits for all 25 manufacturers for scenario 2 are presented in Table 6. It can be observed that although the top five manufacturers account for a 91.40% share of total benefits for the 25 manufacturers the benefits are mainly generated by the GHG emission reductions from the manufacturers of basic metals, of other non-metallic mineral products, and of chemical materials.

**Figure 9:**
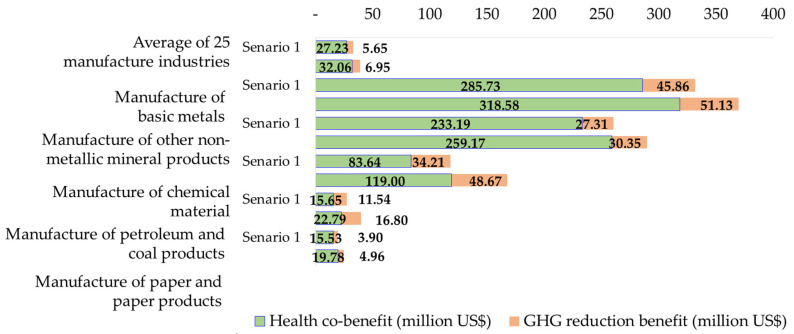
The benefits comparison for the top five groups of manufacturers for the two scenarios.

Figure 9 presents the GHG emission reduction benefits and health co-benefits of air pollution reductions for the top five ranking manufacturers both for scenario 1 and scenario 2. These five manufacturer categories have the same rankings in terms of the total benefits but the GHG reduction benefit and health co-benefit rankings are different from the corresponding total benefit rankings. The results show that the health co-benefits derived from the GHG reduction for manufacturers of basic metals and manufacturers of other non-metallic mineral products are much higher than their corresponding GHG reduction benefits and the multiple of the health co-benefits to the GHG reduction benefits is much higher than the average for all 25 manufacturers. The result indicates that mitigating GHG emissions from these manufacturers is more effective if the resources are limited since they bring not only the largest total benefits but also the largest health co-benefits.

## 4. Conclusions

The simulated results show that the health co-benefits are about 5 times the GHG reduction benefits for both scenarios. This outcome clearly indicates that not accounting for health co-benefits will not only underestimate the full benefits from the reductions of GHGs but will also make it difficult to implement an efficient mitigation policy such as the carbon charge analyzed here. The results obtained for the first scenario are those where the carbon charges are imposed on those 25 manufacturing groups of concern in the industrial sector for their scope 1 and scope 2 GHG emissions and the second scenario is where only scope 1 GHG emissions are imposed and the costs of electricity use are pushed forward by all other manufacturers and all other sectors.

The total benefits for the two scenarios have similar performance along with the change in the carbon charge rate. They all reveal that the higher the charge rate, the higher the GHG reduction benefits, health co-benefits, and the total benefits from summing the two. Both the GHG reduction benefits and health co-benefits are consistently increasing at a decreasing rate over the 2023–2030 period. That is, the total benefits increase 2.93 times, 1.63 times, and 1.37 times when the rates charged increase every other year. As the four levels of charge rate increase from the lowest of US$ 3.3 per ton to the highest of US$ 23.3 per ton, the increment for the rate charged is 3 times, 1.67 times, and 1.39 times moving from the lowest rate to the highest rate in 2023–2030. These results are the same for both scenarios and this indicates that the rate for the carbon charge increases more than the total benefits when the rate comes into existence. This is consistent with the outcome that the higher the rate of the carbon charge, the lower the total benefits generated for every one US$ increase in the carbon charge rate. Once the increased multiple for the rate for the carbon charge is higher than the increased multiple of total benefits or the rate for the carbon charge becomes less effective, more evaluation is required to determine the subsequent carbon charge rate to avoid a potential barrier in smoothly implementing the carbon charge policy.

Moreover, in the second scenario, the 25 manufacturing groups do not pay a carbon charge for their scope 2 emissions, mainly the use of electricity, but they in fact pay a higher electricity price due to the increase in the cost of electricity generation for the energy sector as the result of imposing the carbon charge. Moreover, there are many entities that do not have to pay a carbon charge as their emissions do not exceed 25 thousand tons a year, but they are the users of electricity. Many products from these entities are the inputs of the above major 25 manufacturers. As a result, although those 25 manufacturers are only charged based on their scope 1 direct GHG emissions, it is highly possible for them to encounter a higher electricity price and a higher cost of inputs from other sectors or manufacturers in the industrial sector as the price of electricity is transferred by the energy sector. For these 25 manufacturer groups, their total burdens, either through the payment of carbon charge or through higher input prices, might be much more expensive than those charged directly by their emissions for scope 1 and scope 2 emissions.

This result is not consistent with the intuition that carbon charges on both direct and indirect scope GHG emissions reduction should generate higher benefits as the control agency expects in Taiwan. However, the results of any type of benefit based on the indirect emissions only largely rely upon the transferability of the electricity price. The predicted results will exist only if the energy sector fully transfers its cost increases due to the carbon charge. However, the largest electricity supplier, Taipower, is a government-owned monopoly in Taiwan. The electricity price is normally determined politically and it is hard for it to reflect the cost of electricity generation. Thus, in the real world, the second scenario may not contribute higher GHG emission reduction benefits, health co-benefits, or total benefits when including both as the results obtained in this study would suggest. Even if the electricity price increases, its transferability to all other sectors and the 25 manufacturers of concern is different. Thus, one of the limitations of this study is that the second scenario does not account for the classification of the price transferability for different manufacturers and sectors, to reflect better on the effects of electricity price. Such a consideration is another angle deserving to be explored by building upon the results obtained in this study. Another limitation of this study is that it does not include the cost side of a carbon charge at different rates and their impacts on different groups of stakeholders, such as a sector on which the carbon charge is imposed and one that is not.

The validation of the results from the methodology used in this study is ensured as long as the operations follow all the procedures presented in the Section 2.2 and their corresponding details presented in the sequential Section 2.3, Section 2.4, Section 2.4.1 and Section 2.4.2 along with the public information prepared by government related agencies and the data from the existing research. A validity test could involve a considerable number of combinations of variables, parameters, functional forms, or any related data. This is pragmatically infeasible. A new result might be produced at one extreme as everything is changed. However, not much will be changed since all the procedures are comprised in the methodology constructed in this study.

Further studies can be developed from the following directions. The evaluation of monetary health co-benefits in this study involves the use of the value of a statistical life. The latest available existing study in Taiwan is referred to for the purposes of this study. The transferred value of a statistical life only takes into account the differentiation of incomes. However, the value of a statistical life varies not only with income but also the type of occupation. Future studies could further look into having a better evaluation of the value of a statistical life as it is one important component in the evaluation of health co-benefits. From the standpoint of a proper selection of mitigation policy or the determination of the carbon charge rate, this study only evaluates the monetary benefits of the GHG emission reductions and the monetary health co-benefits for different rates of carbon tax. Future research can also evaluate the cost of GHG mitigation through the carbon charge at a specific rate for a full cost-benefit analysis to prepare for the selection of an appropriate policy design.

## Figures and Tables

**Table 1:** The social cost of carbon under different discount rates estimated by the US Interagency Working Group on the social cost of carbon *.

Year	Discount Rate		
	Average SCC under a 5% Discount Rate	Average SCC under a 3% Discount Rate	Average SCC under a 2.5% Discount Rate
2020	14	51	76
2025	17	56	83
2030	19	62	89
2035	22	67	96
2040	25	73	103
2045	28	79	110
2050	32	85	116

Source: [67]. * The unit for all the magnitudes in the Table is US$/tCO_2e_.

**Table 2:** The effect of a one-ton pollution reduction for each pollutant on the concentration from different sources ^1^.

Pollutant	Point Source	Line Source ^2^	Area Source
PM_2.5_	0.00009866	0.00013522	0.00007858
SO_x_	0.00001413	--	0.00002752
NO_x_	0.00000500	0.00000629	0.00004108

Source: [74]. ^1^ The unit for all results is ug/m^3^/ton. ^2^ The simulation result for SO_x_ for the line source is insignificant.

**Table 3:** Population projection for Taiwan in 2023–2030.

Year	Projected Population (Persons)
2023	23,487,421
2024	23,471,823
2025	23,436,816
2026	23,402,062
2027	23,360,315
2028	23,313,038
2029	23,260,030
2030	23,201,540

Source: [78].

**Table 4:** All related parameters for VSL transfer.

Parameter	Value	Definition
$VSL_{2014}^{a}$	11.93	The VSL computed in the study by [75] in 2019; unit: million US$
$\in_{W}^{b}$	0.2476	The earnings elasticity estimated for monthly earnings between US$ 1333.3 and US$ 1600.01
$W_{2014}^{c}$	1333.3	Average monthly earnings in US$ in 2014
$W_{2020}^{c}$	1600.01	Average monthly earnings in US$ in 2020
$CPI_{2014}$	98.93	The consumer price indices ^d^ for 2014 and 2020, with A base year of 2015; i.e., $CPI_{2015}=100$
$CPI_{2020}$	102.55	

^a^ This study uses the study by [75] for reference. ^b^ The VSLs from [75] are used to compute the elasticity results used in this study. ^c^ Data of $W_{2014}$ and $W_{2020}$ are from [79]. ^d^ Data of $CPI_{2014}$ and $CPI_{2020}$ are from [80].

**Table 5:** Health co-benefits and GHG reduction benefits for 25 manufacturers simulated in scenario 1.

Manufacturer	Health Co-Benefits (Million US$)	Share of Health Co-Benefit of Each Manufacturer (%)	GHGs Reduction Benefits (Million US$)	Share of GHG Reduction Benefits of Each Manufacturer (%)	Total Benefits (Million US$)	Share of Total Benefits of Each Manufacturer (%)
Manufacturers of Basic Metals	285.73	41.98	45.86	32.48	331.59	40.34
Manufacturers of Other Non-metallic Mineral Products	233.19	34.26	27.31	19.34	260.50	31.69
Manufacturers of Chemical Materials	83.64	12.29	34.21	24.23	117.85	14.34
Manufacturers of Petroleum and Coal Products	15.65	2.30	11.54	8.17	27.18	3.31
Manufacturers of Paper and Paper Products	15.53	2.28	3.90	2.76	19.43	2.36
Manufacturers of Chemical Products	13.56	1.99	1.56	1.10	15.11	1.84
Manufacturers of Electronic Parts and Components	3.16	0.46	8.06	5.71	11.22	1.37
Manufacturers of Textiles	8.47	1.24	2.13	1.51	10.60	1.29
Manufacturers of Fabricated Metal Products	7.20	1.06	2.84	2.01	10.05	1.22
Manufacture of Plastics Products	4.31	0.63	1.51	1.07	5.82	0.71
Manufacturers of Food Products	4.39	0.65	0.43	0.30	4.82	0.59
Other Manufacturing	1.89	0.28	0.50	0.35	2.39	0.29
Manufacturers of Rubber Products	1.44	0.21	0.38	0.27	1.82	0.22
Manufacturers of Electrical Equipment	0.83	0.12	0.14	0.10	0.97	0.12
Manufacturers of Wood and of Products of Wood and Bamboo	0.74	0.11	0.07	0.05	0.81	0.10
Printing and Reproduction of Recorded Media	0.25	0.04	0.19	0.13	0.44	0.05
Manufacturers of Machinery and Equipment	0.24	0.03	0.18	0.13	0.42	0.05
Manufacturers of Motor Vehicles and Parts	0.19	0.03	0.16	0.11	0.35	0.04
Manufacturers of Other Transport Equipment and Parts	0.09	0.01	0.06	0.04	0.15	0.02
Manufacturers of Computers, Electronic and Optical Products	0.01	0.00	0.09	0.07	0.10	0.01
Manufacturers of Pharmaceuticals and Medicinal Chemical Products	0.06	0.01	0.03	0.02	0.08	0.01
Manufacturers of Leather, Fur and Related Products	0.07	0.01	0.01	0.01	0.08	0.01
Manufacturers of Beverages and Tobacco	0.04	0.01	0.01	0.01	0.05	0.01
Manufacturers of Furniture	0.03	0.00	0.01	0.01	0.05	0.01
Manufacturers of Wearing Apparel and Clothing Accessories	0.00	0.00	0.01	0.01	0.01	0.00
Total benefits of 25 manufacturers	680.71	100.00	141.19	100.00	821.90	100.00
Average for 25 manufacturers	27.23	82.82	5.65	17.18	32.88	100.00

**Table 6:** Health co-benefits and GHG reduction benefits for the 25 manufacturers simulated in scenario 2.

Manufacturer category	Health Co-Benefits (Million US$)	Share of Health Co-Benefit of Each Manufacturer (%)	GHG Reduction Benefits (Million US$)	Share of GHG Reduction Benefits of Each Manufacturer (%)	Total Benefits (Million US$)	Share of Total Benefits of Each Manufacturer (%)
Manufacturers of Basic Metals	318.58	39.75	51.13	29.44	369.71	37.91
Manufacturers of Other Non-metallic Mineral Products	259.17	32.34	30.35	17.47	289.52	29.69
Manufacturers of Chemical Materials	119.00	14.85	48.67	28.02	167.68	17.20
Manufacturers of Petroleum and Coal Products	22.79	2.84	16.80	9.67	39.60	4.06
Manufacturers of Paper and Paper Products	19.78	2.47	4.96	2.86	24.75	2.54
Manufacturers of Chemical Products	16.26	2.03	1.87	1.07	18.12	1.86
Manufacturers of Textiles	12.20	1.52	3.06	1.76	15.26	1.56
Manufacturers of Fabricated Metal Products	8.73	1.09	3.45	1.98	12.18	1.25
Manufacturers of Electronic Parts and Components	3.16	0.39	8.05	4.63	11.21	1.15
Manufacturers of Food Products	7.70	0.96	0.75	0.43	8.46	0.87
Manufacturers of Plastics Products	5.67	0.71	2.00	1.15	7.67	0.79
Other Manufacturing	3.06	0.38	0.81	0.47	3.87	0.40
Manufacturers of Rubber Products	1.88	0.23	0.50	0.29	2.38	0.24
Manufacturers of Electrical Equipment	1.10	0.14	0.18	0.10	1.28	0.13
Manufacturers of Wood and of Products of Wood and Bamboo	1.00	0.12	0.10	0.06	1.09	0.11
Printing and Reproduction of Recorded Media	0.36	0.04	0.27	0.15	0.62	0.06
Manufacturers of Machinery and Equipment	0.29	0.04	0.22	0.13	0.52	0.05
Manufacturers of Motor Vehicles and Parts	0.26	0.03	0.21	0.12	0.48	0.05
Manufacturers of Other Transport Equipment and Parts	0.12	0.01	0.08	0.05	0.20	0.02
Manufacturers of Pharmaceuticals and Medicinal Chemical Products	0.09	0.01	0.04	0.02	0.13	0.01
Manufacturers of Computers, Electronic and Optical Products	0.01	0.00	0.12	0.07	0.13	0.01
Manufacturers of Leather, Fur and Related Products	0.10	0.01	0.02	0.01	0.12	0.01
Manufacturers of Beverages and Tobacco	0.08	0.01	0.02	0.01	0.10	0.01
Manufacturers of Furniture	0.04	0.01	0.02	0.01	0.06	0.01
Manufacturers of Wearing Apparel and Clothing Accessories	0.00	0.00	0.02	0.01	0.02	0.00
Total benefits of 25 manufacturers	801.43	100.00	173.71	100.00	975.14	100.00
Average for 25 manufacturers	32.06	82.19	6.95	17.81	39.01	100.00

## Data Availability

Data is not publicly available. The data can be obtained on the request of the authors.
